# RORα inhibits gastric cancer proliferation through attenuating G6PD and PFKFB3 induced glycolytic activity

**DOI:** 10.1186/s12935-023-03201-4

**Published:** 2024-01-06

**Authors:** Xiaoshan Wang, Junyi Zhang, Yuwei Wu, Yuqing Zhang, Siyuan Zhang, Angqing Li, Jian Wang, Zhengguang Wang

**Affiliations:** 1 Department of General Surgery, First Affiliated Hospital of Anhui Medical University, Hefei, 230032 Anhui People’s Republic of China; 2 Department of Occupational Health and Environmental Hygiene, School of Public Health, Anhui Medical University, Hefei, Anhui People’s Republic of China

**Keywords:** RORα, Proliferation, G6PD, PFKFB3, Glycolysis, Gastric cancer

## Abstract

**Background:**

Glycolysis is critical for harvesting abundant energy to maintain the tumor microenvironment in malignant tumors. Retinoic acid-related orphan receptor α (RORα) has been identified as a circadian gene. However, the association of glycolysis with RORα in regulating gastric cancer (GC) proliferation remains poorly understood.

**Methods:**

Bioinformatic analysis and retrospective study were utilized to explore the role of RORα in cell cycle and glycolysis in GC. The mechanisms were performed in vitro and in vivo including colony formation, Cell Counting Kit-8 (CCK-8), Epithelial- mesenchymal transition (EMT) and subcutaneous tumors of mice model assays. The key drives between RORα and glycolysis were verified through western blot and chip assays. Moreover, we constructed models of high proliferation and high glucose environments to verify a negative feedback and chemoresistance through a series of functional experiments in vitro and in vivo.

**Results:**

RORα was found to be involved in the cell cycle and glycolysis through a gene set enrichment analysis (GSEA) algorithm. GC patients with low RORα expression were not only associated with high circulating tumor cells (CTC) and high vascular endothelial growth factor (VEGF) levels. However, it also presented a positive correlation with the standard uptake value (SUV) level. Moreover, the SUV_max_ levels showed a positive linear relation with CTC and VEGF levels. In addition, RORα expression levels were associated with glucose 6 phosphate dehydrogenase (G6PD) and phosphofructokinase-2/fructose-2,6-bisphosphatase (PFKFB3) expression levels, and GC patients with low RORα and high G6PD or low RORα and high PFKFB3 expression patterns had poorest disease-free survival (DFS). Functionally, RORα deletion promoted GC proliferation and drove glycolysis in vitro and in vivo. These phenomena were reversed by the RORα activator SR1078. Moreover, RORα deletion promoted GC proliferation through attenuating G6PD and PFKFB3 induced glycolytic activity in vitro and in vivo. Mechanistically, RORα was recruited to the G6PD and PFKFB3 promoters to modulate their transcription. Next, high proliferation and high glucose inhibited RORα expression, which indicated that negative feedback exists in GC. Moreover, RORα deletion improved fluorouracil chemoresistance through inhibition of glucose uptake.

**Conclusion:**

RORα might be a novel biomarker and therapeutic target for GC through attenuating glycolysis.

**Supplementary Information:**

The online version contains supplementary material available at 10.1186/s12935-023-03201-4.

## Introduction

Gastric cancer (GC) is the fourth leading cause of cancer-associated death worldwide [1]. However, the incidence rates in Europe and America are generally lower than those in Asia and Africa. The incidence of young adults exhibits a progressive rise in both high and low risk regions due to *Helicobacter Pylori* infection, genetic risk factors and poor lifestyles [1]. Unfortunately, most patients tend to suffer an advanced stage despite the development of diagnostic technology [1, 2]. These points indicate that we still lack sufficient knowledge regarding the mechanism of GC. Therefore, it is critical and urgent to discover novel biomarkers and molecular pathways in GC.

Normal cells that do not need extra energy during oxidative phosphorylation exclude tumor cells. Aerobic glycolysis can obtain more energy to maintain rapid proliferation of tumor cells, which causes a reprogramming process of metabolism [3]. Under this kind of requirement. Tumor cells activate various transport proteins and terminated limitation by key enzymes [3]. In previous studies, abnormal glycolysis was shown to play an essential role in tumors through the modulation of clock genes [4–6]. However, the exact clarification of the mechanism between clock genes and glycolysis remains poorly understood.

Retinoic acid-related orphan receptor α (RORα) is widely distributed in mammals to modulate the transcription of target genes in the nucleus. It tends to exhibit different specificities during the complicated process of physiology including lipid, cholesterol metabolism and immune system. [7]. Thus, the dysregulation of RORα is associated with multiple cancers according to previous studies [8–11]. However, few studies between RORα and aerobic glycolysis in tumor. Recent studies have demonstrated RORα reprograms glucose metabolism in glutamine-deficient hepatoma cells, but also inhibits aerobic glycolysis activity in hepatoma cells treated with the RORα activator SR1078 by reducing the expression of pyruvate dehydrogenase kinase 2 (PDK2), inhibiting the phosphorylation of pyruvate dehydrogenase and shifting pyruvate to complete oxidation [12]. Moreover, Glucose 6 phosphate dehydrogenase (G6PD) and phosphofructokinase-2/fructose-2,6-bisphosphatase (PFKFB3) genes were inducers in downstream signal pathways to promote the progression of GC [13–15]. Whether RORα is involved in glucose metabolism through the modulation of G6PD and PFKFB3 in GC is not clear.

Accordingly, we found RORα inhibits GC proliferation and glycolysis through a series of functional experiments in vitro and in vivo. More importantly, RORα was recruited to the promotors of G6PD and PFKFB3 genes to modulate its transcription, thereby, inhibiting GC proliferation and glycolysis. Moreover, the environment with high proliferation and high glucose modulated a negative feedback and inhibited RORα expression in GC. In addition, RORα deletion improved fluorouracil chemoresistance through inhibition of glucose uptake in GC. These findings provide a perspective on the role of RORα in GC.

## Methods

### Bioinformatics analysis

The gene set enrichment analysis (GSEA) algorithm was applied to identify functions and pathways through Reactome database (http://www.reactome.org) and Wikipathways database (http://www.wikipathways.org).

### Patients and specimens

GC patients (pathological diagnosis) were received circulating tumor cells (CTC), vascular endothelial growth factor (VEGF) examination and were collected paraffin-embedded sections in The First Affiliated Hospital of Anhui Medical University (Hefei, Anhui) from 2021 to 2023. The clinicopathological stage was assessed by chest, abdomen and pelvis enhanced CT or MRI according to 7th Edition of the International Union against Cancer tumour–node–metastasis (TNM) classification [16]. The disease-free survival (DFS) time was defined as the time of recurrence locally, distant metastasis or up to 18 months. 18F-FDG PEC/CT examination, imaging diagnosis and standard uptake value (SUV) level were performed and were analyzed by three radiologists. The gender of male 116/71.6% and female 46/28, 4%. Age from 34 to 50 (21/13.0%), 51–60 (55, 14.0%) to 61–88 (86, 53.0%) and median was 63. The details of data collection was illustrated through the patient profile (Additional file 1: Fig. S1). All enrolled patients signed the informed consent which was approved by the human Ethics Committee of Anhui Medical University (20,180,344, Hefei, Anhui).

### Cell culture and treatment

Human GC cell lines (AGS and MKN-74) and Mouse Forestomach Carcinoma (MFC) cell line were purchased from the Procell Life Science and Technology (Wuhan, China). These cells were cultured in RPMI 1640 medium (Procell Life Science and Technology, Wuhan, China) with 10% fetal bovine serum (Thermo Fisher Scientific, Waltham, MA, USA). Culture medium was changed every 2 days. Cells were pretreated with SR1078 (RORα activator, GC16392, GlpBio, Montclair, CA, USA), 3PO (3-(3-pyridinyl)-1-(4-pyridinyl)-2-propen-1-one, PFKFB3 inhibitor, MCE, USA), DHEA (Dehydroepiandrosterone, G6PD inhibitor, MCE, USA) and glucose for 24 h. Cells were pretreated with TGF-β1 (Transforming growth factor beta 1, Cayman chemical, USA) for 48 h. SR1078 was dissolved in phosphate buffer saline (PBS). 3PO, DHEA, TGF-β1 and fluorouracil (GC14466, GlpBio, Montclair, CA, USA) were dissolved in dimethyl sulfoxide (DMSO).

### CRISPR-Cas9 gene deletion

Single guide RNA (sgRNA) oligonucleotides were cloned into LV-U6-spsgRNA(RORα)-CMV-SV40-NLS-spCas9-NLS-Flag-P2A-Puro-T2A-EGFP-WPRE (human) and packaged as lentivirus (BrainVTA, Wuhan, China). The alignment of the DNA sequences of human and mouse RORα genes showed 91% similarity according to NCBI. Herein, The AGS, MKN-74 and MFC cells were infected by virus expressing Cas9 and gRNA at 12 h. The media was resuspended with 2 μg/ml puromycin for 72 h and was verified by western blot to detect transfection efficiency. The RORα-KO sgRNA sequence (human) was GTAATCGACAGTGTTGGCAG.

### Colony formation assay

Cells were seeded into 6-well (2000 cells/well) plates through repeating dilution and were cultured in incubator. The plates were collected to stain with crystal violet after 2 weeks. These colonies were counted to analyze the ability of proliferation.

### CCK-8 assay

Cells were seeded into 96-well tissue microplates (5000 cells/well) to culture according to concentration and time gradient in incubator. The wells were incubated with Enhanced Cell Counting Kit-8 (CCK-8, 10 µl, C0042, Beyotime institute of biotechnology, Haimen, China) for 1 h. The Optical density (OD) values were measured at 450 nm using microplate reader (Thermo Scientific, Inc., USA) to represent the relative cell viability.

### Quantitative real-time PCR (q-PCR)

Total RNA was isolated using TRIzol (Invitrogen, USA) according to the manufacturer’s instructions. The cDNA was generated using a Transcriptor first-strand cDNA synthesis kit (TaKaRa, Shiga, Japan) at 42 ℃ for 40 min and 85 ℃ for 5 min. The primers were showed in Table 1. The q-PCR was performed using the Thermo Biosysterm7500, 96 real-time PCR detection system with TaKaRa SYBR^®^ Green supermix (TaKaRa, Shiga, Japan) according to the manufacturer’s instructions. Every sample obtained a cycle threshold (Cq) value to determine the relative mRNA levels through 2^−ΔΔCt^ method [17]. The β-actin was used as a control for normalization.

**Table 1:** Primer Sequences for q-PCR assay

E-cadherin	Forward	5′–TGC CCA GAA AAT GAA AAA GG–3′
	Reverse	5′–GTG TAT GTG GCA ATG CGT TC–3′
N-cadherin	Forward	5′–GGT GGA GGA GAA GAA GAC CAG–3′
	Reverse	5′–GGC ATC AGG CTC CAC AGT G–3′
Vimentin	Forward	5′–GAG AAC TTT GCC GTT GAA GC–3′
	Reverse	5′–GCT TCC TGT AGG TGG CAA TC–3′
G6PD	Forward	5′–AAA CGG TCG TAC ACT TCG GG–3′
	Reverse	5′–GGT AGT GGT CGT TGC GGT AG–3′
PFKFB3	Forward	5′–CAG TTG TGG CCT CCA ATA TC–3′
	Reverse	5′–GGC TTC ATA GCA ACT GAT CC–3′
β-actin	Forward	5′–CAT GTA CGT TGC TAT CCA GGC–3′
	Reverse	5′–CTC CTT AAT GTC ACG CAC GAT–3′

Quantitative real-time PCR, q-PCR

### Western blot

The protein lysate was obtained from cells or samples through lysis buffer dissolution, and was quantified by BCA Protein kit (Santa Cruz Biotechnology, USA). The target proteins were separated by 10% SDS-PAGE and transferred to polyvinylidene fluoride (PVDF) membrane. Subsequently, the membrane was blocked and was incubated with primary RORα (Rabbit, DF3196, 1:1000 dilution, Affinity Biosciences, Beijing, China), G6PD (Rabbit, GTX101218, 1:1000 dilution, GeneTex, USA), PFKFB3 (Rabbit, ab181861, 1:2000 dilution, Abcam, USA) and β-actin (Rabbit, 1:1000 dilution, Abcam, USA) antibodies for 24 h at 4 ℃, respectively. After washing three times with TBST. The membrane was incubated with conjugated second antibody for 2 h at room temperature. Finally, the bands were imaged by chemiluminescence system (Tanon, Shanghai, China).

### Immunohistochemical staining

The paraffin-embedded sections were deparaffinized and hydrated with a series of xylene and different concentration of ethyl alcohol. The sections were incubated with 3% H_2_O_2_ for 30 min and then were antigen retrieved by microwave. The primary RORα (1:100 dilution), G6PD (1:1000 dilution), PFKFB3 (1:100 dilution), Ki-67 (Rabbit, 12202 T, 1:400 dilution, Cell Signaling Technology, USA) and Proliferating Cell Nuclear Antigen (PCNA, Rabbit, 13110 T, 1:4000 dilution, Cell Signaling Technology, USA) antibodies were employed to incubate for 2 h at room temperature, respectively. Subsequently, the sections were incubated with the conjugated second antibody for 30 min at room temperature. The DAB kit (ZSGB-BIO, OriGene Technologies, Beijing, China) and 20% hematoxylin were utilized to staining at room temperature. The calculation of relative protein expression levels, and definition of low and high expression levels were mentioned in our previous studies [18].

### Establishment of the GC proliferation mice model

MFC (1 × 10^7^) cells were injected into the subcutaneous flank of 6–8 weeks-old mice (C57, Vital River Laboratory Animal Technology, Beijing, China). When the tumor volume reached 100 to 300 mm^3^. Partial mice were sacrificed to perform immunohistochemistry. Another part of mice treated with fluorouracil (100 mg/kg/w) subsequently through injecting into subcutaneous tissues around the tumor for 4 weeks. These studies complied with international protective guidelines for laboratory animals and ethical standards. This project was approved by the Ethics Committee of Anhui Medical University (20,180,365).

### Chromatin immunoprecipitation (Chip)-qPCR assay

The Chip assay was performed using Chip assay kit (P2078, Beyotime, China). The cells were crosslinked by 37% methanol and terminated by glycine solution (10X) at room temperature. After washing with PBS twice, cells were collected and were sufficiently lysed with SDS lysis buffer. The ultrasonic (VCX750, Sonics, USA) was performed to cut DNA fragments from 200 to 800 bp at 25% power, 4.5 s shock and 9 s clearance for 14 times. The samples were centrifuged and collected supernatant solution to dilute with dilution buffer. Then, protein A + G Agarose was added. After centrifuging the mixture. The new supernatant solution was collected and primary RORα (1 μg; Rabbit; ab278099; Abcam, USA) body was added to immune complex. After a series of centrifugation and washing. The precipitation was collected to perform the q-PCR assay.

### Glycolysis assay

The extracellular acidification rate (ECAR) was performed by Seahorse XFe24 analyzer (Seahorse Bioscience, USA) according to the manufacturer’s guidelines. The sensors were immersed in calibrant to hydrate overnight before assay. Cells were seeded into 24 well plate, and the medium was added to a final volume of 250 µl. Next, cells were washed twice and the XF test medium was added to a final volume of 500 µl. Subsequently, The glucose (10 mM), oligomycin (1 μM), and 2-DG (100 mM) were added sequentially to measure the ECAR levels.

### Statistical analysis

SPSS 19.0 and GraphPad Prism 10.0.0 software were performed to statistical analysis. Chi-square test was utilized to analyze the association of RORα, G6PD and PFKFB3 expression levels. Comparisons between different groups was performed using Pearson correlation analysis, *t*-test or two-way ANOVA. Survival analysis was performed by the Kaplan–Meier method and log-rank test. P value < 0.05 was considered as statistically significant.

## Results

### RORα is negatively associated with GC proliferation and glycolysis

**Fig. 1:**
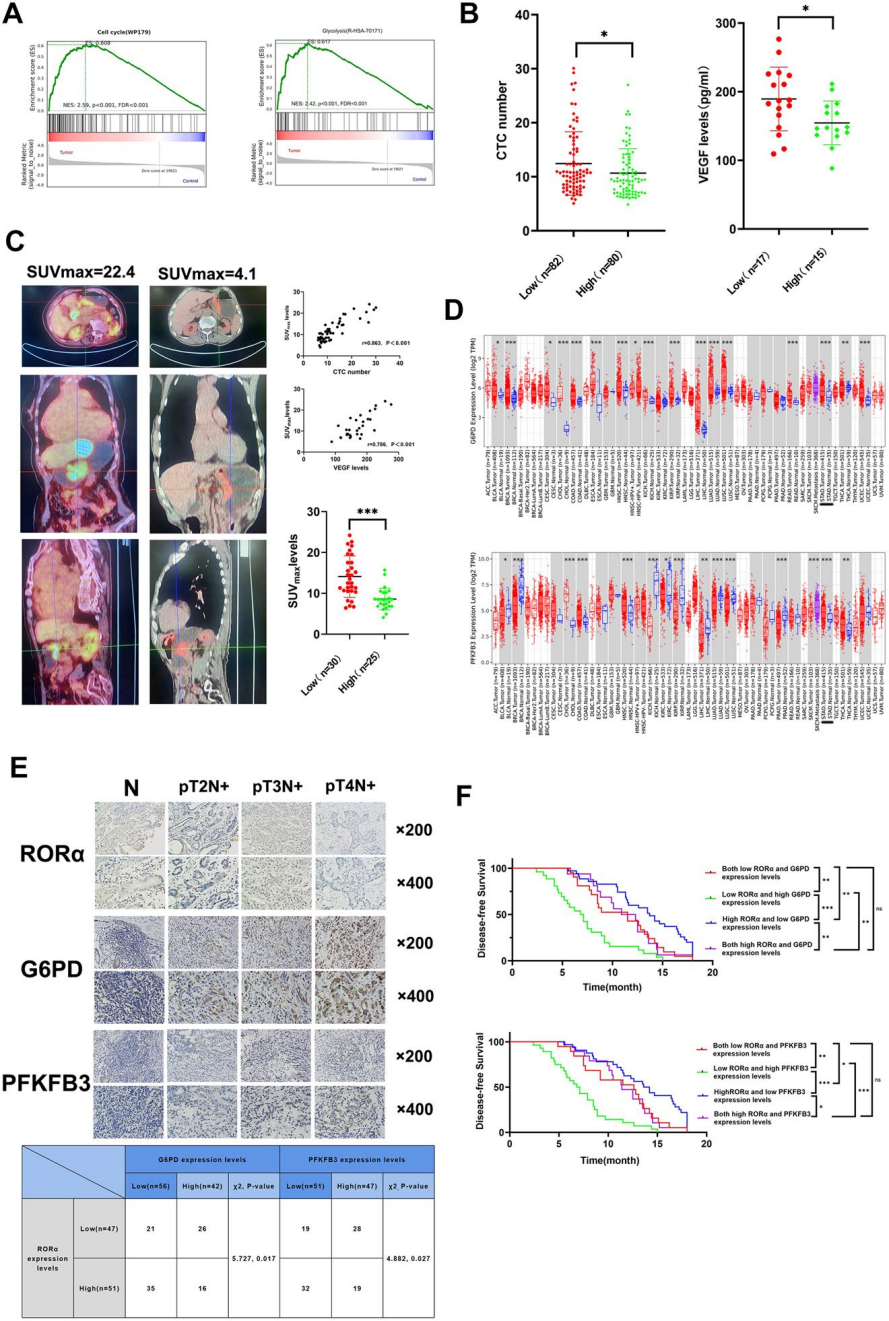
RORα is associated with GC proliferation and glycolysis. **A** GSEA algorithm analysis according to Reactome and Wikipathways database, respectively. **B** The difference analysis of CTC number and VEGF levels in RORα-high and RORα-low groups. **C** Left: Representative 18F-FDG PET/CT images of GC patients with maximum and minimum SUV_max_ levels. Up: The correlation analysis of SUV_max_ levels with CTC number and VEGF levels in GC patients, respectively. Down: The difference analysis of SUV_max_ levels in RORα-high and RORα-low groups. **D** G6PD and PFKFB3 mRNA expression levels in stomach adenocarcinoma-tumor (STAD-tumor) tissues and stomach adenocarcinoma-normal (STAD-normal) tissues from TCGA database were analyzed through TIMER 2.0. **F** Up: RORα, G6PD and PFKFB3 expression levels were detected through immunohistochemistry stain in normal gastric and GC patients with pT2N+, pT3N+ and pT4N+ stages tissues. Original 200 and 400 magnification. Scale bar =100 μm. Down: The correlation analysis of RORα expression levels with G6PD and PFKFB3 expression levels in GC patients, respectively. **G** The DFS time of GC patients with different RORα, G6PD and PFKFB3 expression patterns. All data are represented as the mean ± standard deviation. *P < 0.05, **P < 0.01, and ***P < 0.001

To investigate the involvement of RORα in cell proliferation and glycolysis in GC. GSEA algorithm according to Reactome and Wikipathways database indicated the modulation of RORα was associated with cell cycle and glycolysis (Fig. 1A). To further verify the role of RORα in GC. We collected large amount of clinicopathological data to conduct a retrospective study. First, CTC number and VEGF levels as common monitoring indices were employed to implicate the proliferation of tumors in clinical work and oncology research [19, 20]. Thus, we found GC patients with low RORα expression levels tend to obtain high CTC number and VEGF levels (Fig. 1B). Second, 18F-FDG PEC/CT was utilized to detect glucose uptake due to the characteristics of tumor (Fig. 1C). The result revealed GC patients with low RORα expression levels also tended to obtain high SUVmax levels. Third, the analysis of SUVmax levels with CTC and VEGF levels showed a positive correlation which indicated cancer cells often take advantage of glycolysis to support abnormal proliferation (Fig. 1C**)**. To explore the key drives between RORα and glycolysis.

Glycometabolism-associated genes, G6PD and PFKFB3, were selected for exploration based on previous studies [13–15]. TIMER 2.0 (http://timer.cistrome.org/) verified abnormal expression patterns from The Cancer Genome Atlas Program (TCGA) database in GC (Fig. 1D). Furthermore, RORα expression levels showed a negative correlation with G6PD and PFKFB3 expression levels in GC patients (Fig. 1E). Additionally, GC patients with low RORα and high G6PD or low RORα and high PFKFB3 expression patterns exhibited the poorest DFS compared to other expression patterns (Fig. 1F). In summary, these results indicate that RORα plays a crucial role in gastric cancer proliferation and glycolysis by modulating G6PD and PFKFB3 genes.

### RORα deletion promotes GC proliferation and is reversed by SR1078

**Fig. 2:**
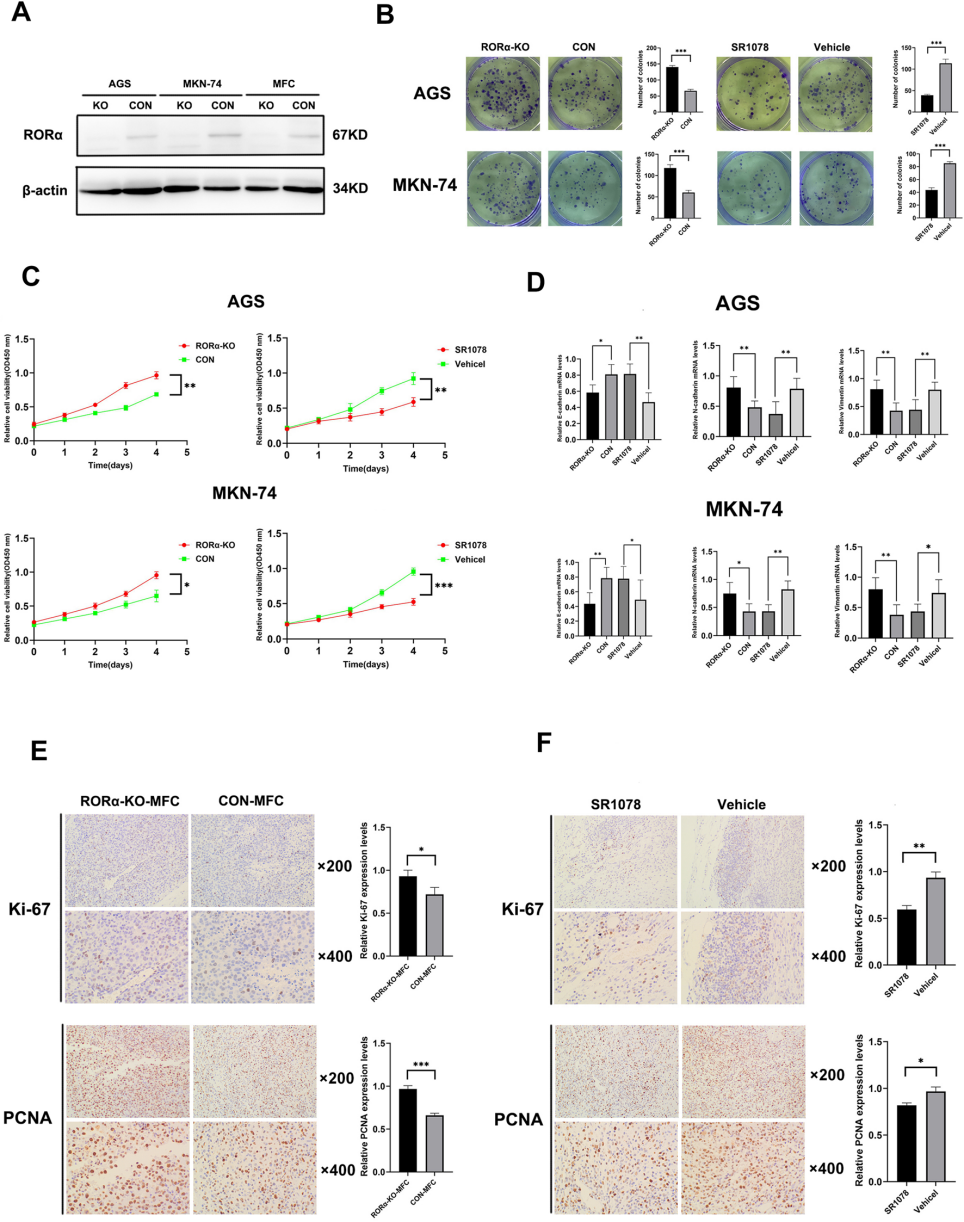
RORα deletion promotes GC proliferation and is reversed by SR1078. **A** RORα expression levels were detected by western blot in lentiviral transfected AGS, MKN-74 and MFC cells. β-actin as a loading control. **B** The colonies of GC cells were performed with colony formation assay. N = 3. **C** The absorbance value of RORα-KO GC cells or GC cells treated with SR1078(5 µM) according to time gradient through CCK-8 assay. N = 3. **D** The relative mRNA levels of E-cadherin, N-cadherin and Vimentin were detected by qPCR assay in RORα-KO GC cells or GC cells treated with SR1078(5 µM). N=3. **E** The expression levels of Ki-67 and PCNA were detected through immunohistochemistry stain and MOD method in subcutaneous tumor. Original 200 and 400 magnification. Scale bar = 100 μm. N = 3. The RORα-KO-MFC cells were injected into subcutaneous flank of mice until the tumor volume reached 100 to 300 mm^3^. **F** The expression levels of Ki-67 and PCNA were detected through immunohistochemistry stain and MOD method in subcutaneous tumor. Original 200 and 400 magnification. Scale bar = 100 μm. N = 3. The MFC cells treated with SR1078 (5 µM) were injected into subcutaneous flank of mice until the tumor volume reached 100 to 300 mm^3^. All data are represented as the mean ± standard deviation. Vehicle was PBS. *P < 0.05, **P < 0.01, and ***P < 0.001

Despite the reduction in RORα expression observed in our previous study on GC, its potential influence on the results of functional experiments cannot be overlooked due to its high basal expression [18]. Thus, the technology of CRISPR-Cas9 gene deletion was employed to kickout RORα gene from AGS, MKN-74 and MFC cells through the verification of western blot (Fig. 2A). Moreover, SR1078 as a RORα activator was identified to the function of tumor suppression [12]. Next, the colony formation, CCK-8, and epithelial-mesenchymal transition (EMT) were performed to investigate whether RORα affects GC proliferation. The RORα-KO group significantly increased colony formation, OD levels, and N-cadherin and Vimentin mRNA levels, while decreasing E-cadherin mRNA levels compared to the CON group in GC cells (Fig. 2B–D). However, these phenomena were reversed by SR1078 (Fig. 2E). Moreover, the subcutaneous tumor group of RORα-KO-MFC showed higher Ki-67 and PCNA expression levels than that of CON-MFC group in mice, while these phenomena were also reversed by SR1078 (Fig. 2F).

### RORα deletion promotes glycolysis and is reversed by SR1078 in GC cells

**Fig. 3:**
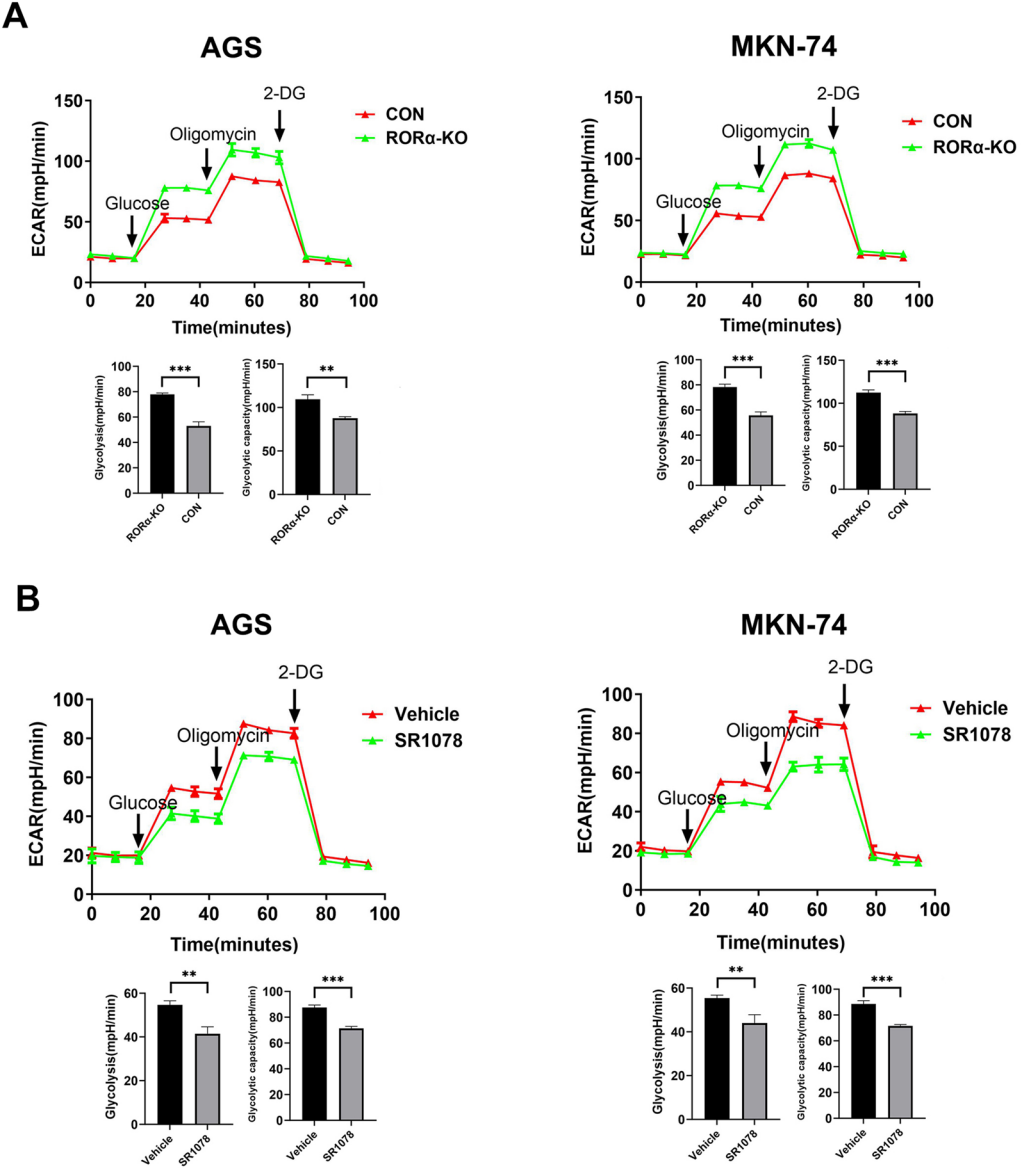
RORα deletion promotes glycolysis and is reversed by SR1078 in GC cells. **A** The changes of ECAR levels in RORα-KO GC cells. The glycolysis and glycolytic capacity were measured in the treatment of glucose and oligomycin, respectively. N = 3. **B** The changes of ECAR levels in GC cells treated with SR1078(5 µM). The glycolysis and glycolytic capacity were measured in the treatment of glucose and oligomycin, respectively. N = 3. All data are represented as the mean ± standard deviation. Vehicle was PBS. *P < 0.05, **P < 0.01, and ***P < 0.001

To investigate the role of RORα in aerobic glycolysis of GC cell, we measured the ECAR levels in RORα-KO GC cells or GC cells treated with SR1078. The RORα-KO GC cells enhanced glycolysis and glycolytic capacity (Fig. 3A). However, these phenomena were reversed in GC cells treated with SR1078 (Fig. 3B**)**. Therefore, RORα expression affects aerobic glycolysis in GC cells.

### RORα deletion promotes glycolysis induced by 3PO and DHEA in GC cells

**Fig. 4:**
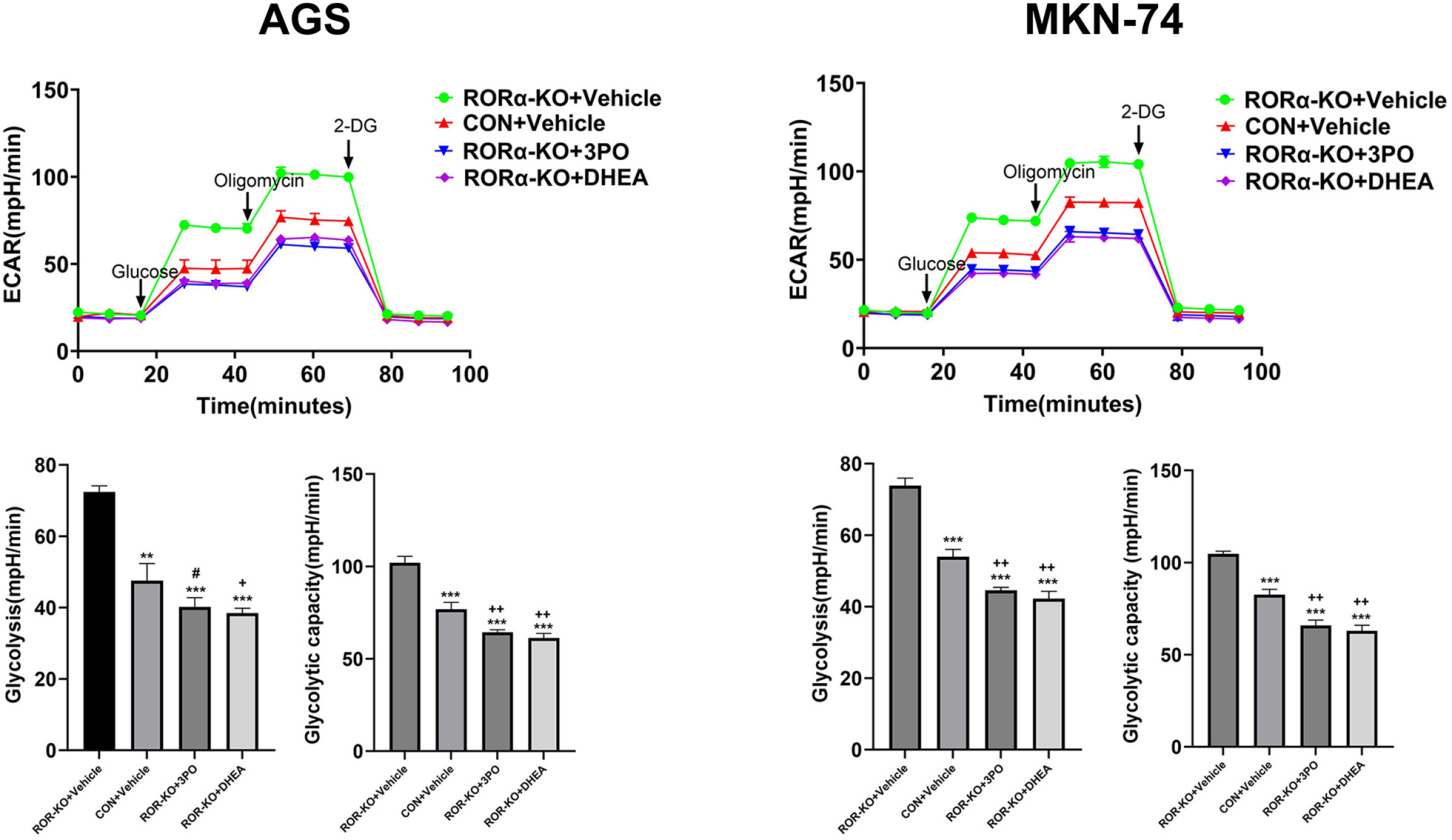
RORα deletion promotes glycolysis induced by 3PO and DHEA in GC cells The changes of ECAR levels in RORα-KO GC cells treated with 3PO (25 µM) or DHEA (250 µM). The glycolysis and glycolytic capacity were measured in the treatment of glucose and oligomycin, respectively. N = 3. All data are represented as the mean ± standard deviation. Vehicle was DMSO. *P < 0.05, **P < 0.01, and ***P < 0.001. Vs. RORα-KO+Vehicle group. ^+^P < 0.05, ^++^P < 0.01, and ^+++^P < 0.001. Vs. CON+Vehicle group

To further verify whether G6PD and PFKFB3 are the key drives among RORα and glycolysis. We selected its inhibitors 3PO and DHEA to explore mechanism of modulation. In previous studies, 3PO and DHEA were classical inhibitors to be employed to explore the pathways of PFKFB3 and G6PD associated glycolysis in oncogenesis [21–24]. ECAR measurements showed RORα-KO plus 3PO or RORα-KO plus DHEA group obviously offset promotive effect of RORα-KO plus Vehicle group on glycolysis and glycolytic capacity in GC cells (Fig. 4**)**. These results indicate RORα affects glycolytic activity through the mediation of G6PD and PFKFB3.

### RORα deletion promotes GC proliferation induced by 3PO and DHEA

**Fig. 5:**
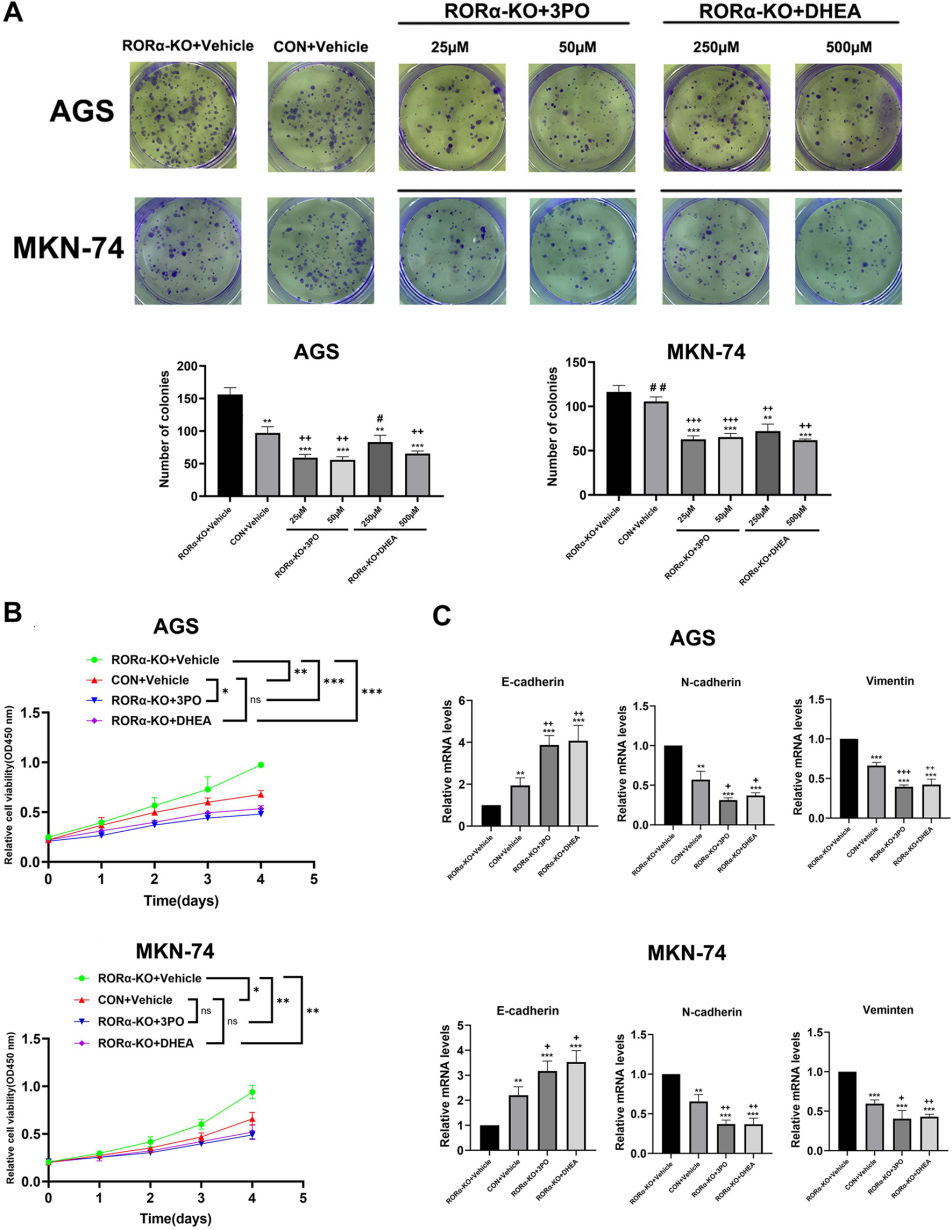
RORα deletion promotes GC proliferation induced by 3PO and DHEA in vitro. **A** The colonies of GC cells were performed with colony formation assay. N=3. ^#^P > 0.05. Vs. CON+Vehicle group in AGS cell. ^##^P > 0.05. Vs. RORα-KO+Vehicle group in MKN-74 cell. **B** The absorbance value of RORα-KO GC cells treated with 3PO (25 µM) or DHEA (250 µM) according to time gradient through CCK-8 assay. N = 3. **C** The relative mRNA levels of E-cadherin, N-cadherin and Vimentin were detected by qPCR assay in RORα-KO GC cells treated with 3PO (25 µM) or DHEA (250 µM). N = 3. All data are represented as the mean ± standard deviation. Vehicle was DMSO. *P < 0.05, **P < 0.01, and ***P < 0.001. Vs. RORα-KO+Vehicle group. ^+^P < 0.05, ^++^P < 0.01, and ^+++^P < 0.001. Vs. CON+Vehicle group

We suggest RORα affects GC proliferation which is also associated with G6PD and PFKFB3 expression. The colony formation, CCK-8, and EMT showed RORα-KO plus 3PO or RORα-KO plus DHEA group obviously offset promotive effect of RORα-KO plus Vehicle group on proliferation in GC cells (Fig. 5A–C). Moreover, the subcutaneous tumor group of RORα-KO plus 3PO or RORα-KO plus DHEA revealed a reduction of Ki-67 and PCNA expression levels compared with RORα-KO plus Vehicle group (Additional file 2: Fig. S2).

### RORα inhibits G6PD and PFKFB3 gene expression through the recruitment of promoters in GC cells

**Fig. 6:**
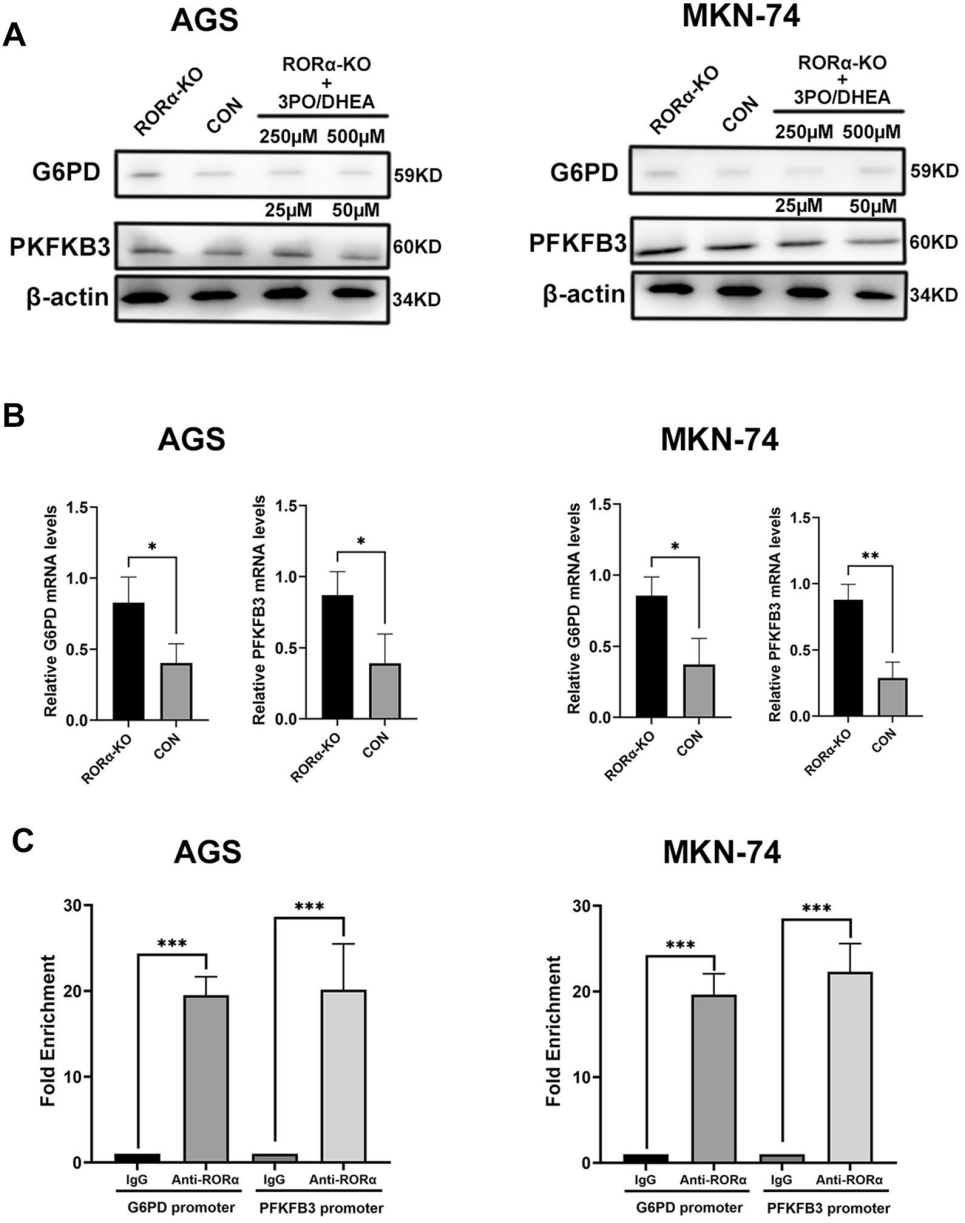
RORα inhibits G6PD and PFKFB3 gene expression through the recruitment of promoter in GC cells. **A** G6PD and PFKFB3 protein expression levels were detected by western blot in RORα-KO, CON, RORα-KO plus 3PO and RORα-KO plus DHEA GC cells. β-actin as a loading control. **B** G6PD and PFKFB3 mRNA levels were detected by q-PCR in lentiviral transfected GC cells. N = 3. **C** RORα was recruited to G6PD and PFKFB3 gene promoter in GC cells through Chip assay. N=3. All data are represented as the mean ± standard deviation. *P < 0.05, **P < 0.01, and ***P < 0.001

To explore the potential mechanism of RORα modulation to G6PD and PFKFB3 genes in GC. The western blot bands showed G6PD and PFKFB3 expression was enhanced in RORα-KO compared with CON GC cells, while 3PO and DHEA offset this enhancement (Fig. 6A). These results suggested the existence of an RORα/G6PD/PFKFB3 regulatory axis. Next, the q-PCR assay showed RORα could inhibit G6PD and PFKFB3 mRNA levels in GC cells (Fig. 6B). Subsequently, the Chip assay was employed to explore the deep mechanism. We found RORα is recruited to the promoters of G6PD and PFKFB3 genes (Fig. 6C). Taken together, RORα was recruited to promoter of G6PD and PFKFB3 gene so that to weaken its ability of transcription,

### High proliferation and high glucose inhibit RORα expression in GC

**Fig. 7:**
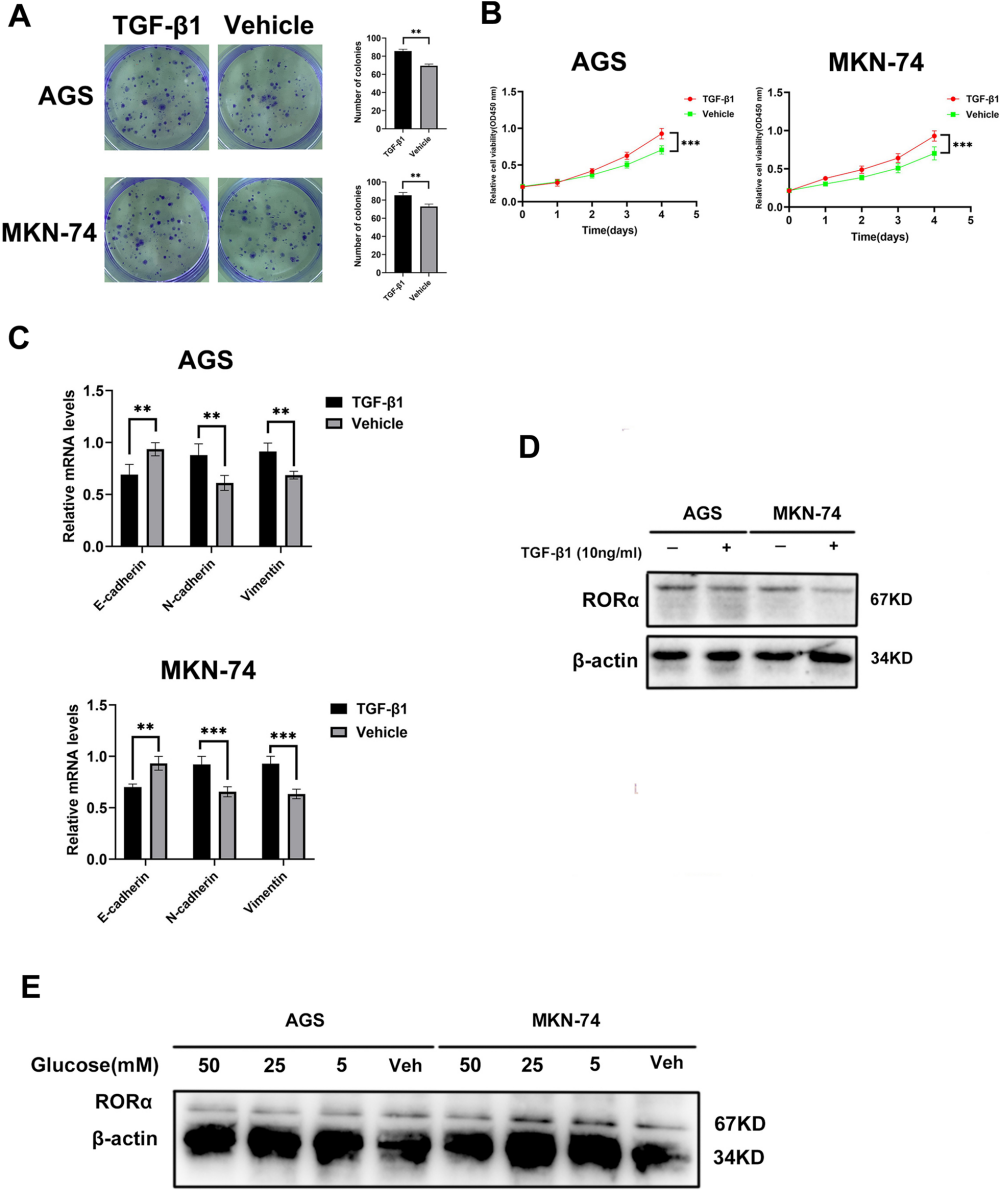
High proliferation and high glucose inhibit RORα expression in GC cells. **A** The colonies of GC cells treated with TGF-β1 (10ng/ml) were performed with colony formation assay. N = 3. **B** The absorbance value of GC cells treated with TGF-β1 (10 ng/ml) according to time gradient through CCK-8 assay. N = 3. **C** The relative mRNA levels of E-cadherin, N-cadherin and Vimentin were detected by qPCR assay in GC cells treated with TGF-β1 (10 ng/ml). N = 3. **D** RORα protein expression levels were detected by western blot in GC cells treated with TGF-β1 (10 ng/ml). **E** RORα protein expression levels were detected by western blot in GC cells treated with glucose in concentration gradient. All data are represented as the mean ± standard deviation. Vehicle was DMSO. β-actin as a loading control. *P < 0.05, **P < 0.01, and ***P < 0.001

We constructed an environment with high proliferation or high glucose to verify an existence of negative feedback. The GC cells treated with TGF-β1 enhanced the colonies, OD levels and N-cadherin and Vimentin mRNA levels, and weakened E-cadherin mRNA levels through colony formation, CCK-8, and EMT assays, respectively (Fig. 7A–C). Thus, GC cells with high proliferation obviously inhibited RORα expression through western blot bands (Fig. 7D). Moreover, we utilized glucose to culture GC cells and found RORα expression was reduced in high glucose group in a concentration dependent gradient (Fig. 7E). Next, we further constructed an in vivo model through the injection of MFC cells treated with TGF-β1 or glucose. The subcutaneous tumor group of TGF-β1 showed higher Ki-67 and PCNA expression levels than that of Vehicle group in mice (Additional file 3: Fig. S3A). The western blot bands showed reduction of RORα expression in TGF-β1 group (Additional file 3: Fig. S3B). Moreover, The subcutaneous tumor group of glucose showed lower RORα expression levels than that of Vehicle group in mice (Additional file 3: Fig. S3C).

### RORα deletion improved fluorouracil chemoresistance through inhibition of glucose uptake in GC

**Fig. 8:**
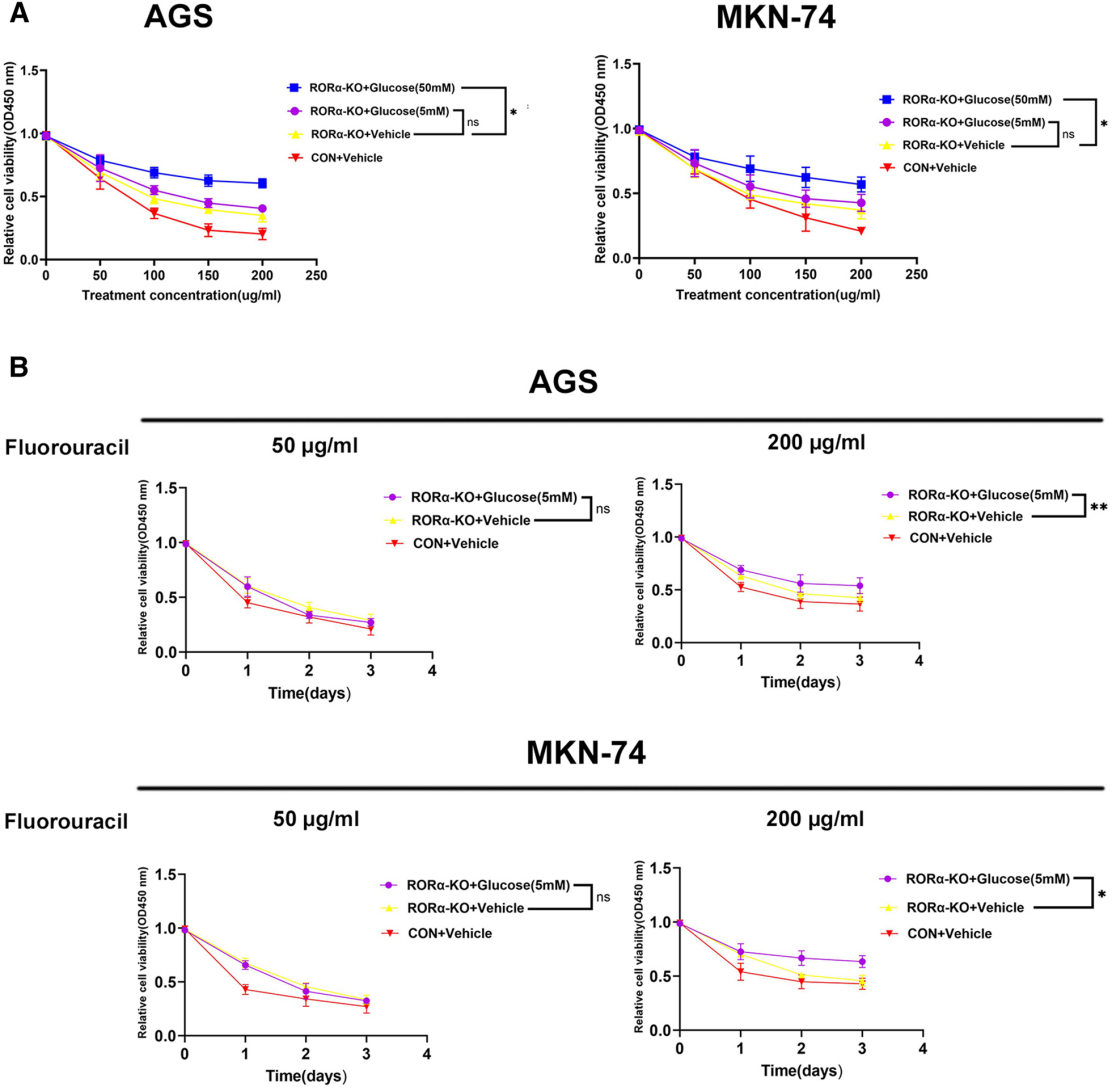
Inhibition of glucose uptake improved fluorouracil chemoresistance in RORα-KO GC cells. **A** The GC cells were pretreated with 5mM or 50mM glucose for 24h, and then were treated with fluorouracil according to 0, 50, 100, 150 and 200 µg/ml concentration gradient. N = 3. **B** The GC cells were pretreated with 5 mM or 50 mM glucose for 24 h, and then were treated with fluorouracil (50 and 200 µg/ml concentration) according to time gradient. N = 3. The absorbance value was measured through CCK-8 assay. All data are represented as the mean ± standard deviation. Vehicle was DMSO. *P < 0.05, **P < 0.01, and ***P < 0.001

Fluorouracil is the first line chemotherapy for GC patients [16]. Glycolysis is associated with enhanced chemoresistance in GC [25]. Therefore, we suggest that inhibition of glucose uptake would sensitize fluorouracil treatment for GC with RORα gene deletion. In vitro and in vivo assays, RORα-KO plus 50 mM glucose group exhibited a significant chemoresistance to fluorouracil compared with RORα-KO plus Vehicle group, while RORα-KO plus 5 mM glucose group did not affect fluorouracil chemoresistance compared with RORα-KO plus Vehicle group (Fig. 8A, Additional file 4: Fig. S4A–C). To further detect the difference between high and low glucose uptake. we utilized 50 and 200 µg/ml concentration fluorouracil to treat GC cells according to time gradient. We found RORα-KO plus 5 mM glucose group still not affect fluorouracil chemoresistance compared with RORα-KO plus Vehicle group under the 50 µg/ml concentration fluorouracil. However, when the condition shifting to 200 µg/ml concentration fluorouracil. RORα-KO plus 5 mM glucose group enhanced chemoresistance to fluorouracil compared with RORα-KO plus Vehicle group with the extension of time (Fig. 8B).

## Discussion

Aerobic glycolysis plays a significant role in the reprogramming tumor microenvironment and eventually results in abnormal proliferation, progression, invasion and metastasis [3]. G6PD and PFKFB3 are key enzymes that participate in glucose metabolism [13–15]. The present study revealed RORα inhibits GC proliferation through attenuating G6PD and PFKFB3 induced glycolytic activity. These findings indicated a novel interaction between RORα and glycolysis in GC.

Reportedly, RORα is one of circadian rhythm genes and is involved in multiple metabolic cycles in mammals, and its dysregulation results in disease and even cancer [8, 10, 26]. In detail, RORα suppressed breast tumor invasion by inducing SEMA3F expression. RORα as a transcription regulator to mediate SEMA3F transcription [8]. Moreover, RORα suppressed EMT of GC cells via Wnt/β-Catenin Pathway [27]. In addition, RORα inhibited hepatocellular carcinoma proliferation, invasion and migration through downregulation of chemokine CXCL5 [28]. Thus, RORα performed an antitumor function and was obtained verification. However, whether RORα modulates aerobic glycolysis to be involved in oncogenesis is still an unknow domain of exploration in GC. In our present study, we attempted to explore the association among RORα and glycolysis through bioinformatics analysis. The GSEA algorithm indicated RORα inhibits GC proliferation and glycolysis according to Reactome and Wikipathways database. Next, we collected abundant clinicopathological data from GC patients combined with a series of functional experiments in vitro and in vivo to verify this indication.

G6PD assists in the metabolism of glucose and is mainly involved in pentose phosphate pathway which induces the occurrence and progression of disease [29]. Its metabolites including glyceraldehyde-3-phosphate and fructose-6-phosphate recycled glycolysis to obtain energy [29]. Recent studies demonstrated the activation of G6PD regulates Warburg effect and affects GC proliferation [15]. G6PD altered aerobic glycolysis and promoted tumor progression via pentose phosphate pathway [30]. PFKFB3 as a glucose regulator and is associated with diabetes and cancer [13, 14, 31, 32]. However, whether G6PD and PKFKB3 are downstream targets to induce glycolysis via RORα is not clear. Thus, we utilized TIMER 2.0 and found G6PD and PFKFB3 expression was increased in GC. Moreover, RORα expression levels revealed a negative correlation with G6PD and PFKFB3 expression levels in GC tissues. GC patients with low RORα and high G6PD or low RORα and high PFKFB3 expression patterns obtained a poorest DFS compared with other patterns. These results indicated a regulatory axis among RORα/G6PD/PFKFB3 in GC. To verify this speculation. We utilized their inhibitors DHEA and 3PO to offset the regulation of RORα in GC proliferation and glycolysis. These phenomena demonstrated RORα inhibits GC proliferation through attenuating G6PD and PFKFB3 induced glycolytic activity. Mechanically, RORα was recruited to the promoters of G6PD and PFKFB3, leading to the regulation of GC proliferation and glycolysis.

It has been reported that TGF-β1 plays an ambiguous role in tumors, where it can inhibit tumor development during the early stages while promoting metastatic spread as the disease progresses [33]. To construct a high proliferation environment, we treated GC or MFC cells with TGF-β1 and verified a viable concentration, consistent with previous report that TGF-β1 induce GC progression [34]. Moreover, we treated GC or MFC cells with glucose in concentration gradient to obtain an optimum environment for GC glycolysis according to previous study [35]. Interestingly, these environments significantly inhibited RORα expression in vitro and in vivo. However, whether this negative feedback is also mediated by G6PD and PFKFB3 was not obtained a further exploration, even would appear other targets to mediate regulation.

Glycolysis enhanced chemoresistance is an ongoing and promising hotpot. In previous studies, the Fibrillin-1/VEGFR2/STAT2 signaling axis promoted cisplatin chemoresistance via modulating glycolysis in ovarian cancer cells [36]. HKDC1 promoted cisplatin, oxaliplatin and fluorouracil chemoresistance through glycolysis in GC [37]. Thus, inhibition of glucose uptake is a viable strategy for cancer therapy through weakening glycolysis-associated chemoresistance [25, 38]. We found RORα deletion barely improve fluorouracil chemoresistance through inhibition of less glucose uptake, while achievement through inhibition of abundant glucose uptake in vitro and in vivo. Of note, when RORα-KO GC cells treated with high concentration fluorouracil, inhibition of less glucose uptake improved fluorouracil chemoresistance with the extension of time. In contrast, RORα-KO GC cells treated with low concentration fluorouracil did not shifting the situation of fluorouracil chemoresistance. These results indicated RORα deletion could improve fluorouracil chemoresistance through inhibition of glucose uptake in GC. Meanwhile, the improvement ability was associated with dependent concentration and time gradient.

However, a few limitations should be mentioned due to objective and subjective reasons. The absence of past medical and therapeutic history might disturb a logically rigorous among interactions of result in GC. To prevent excessive bias and obtain deeply stratified research, further demographic analysis was needed to expand the sample size, extend follow-up time and collect detailed clinicopathological data. Additionally, although we verified a hypothesis that RORα regulates GC proliferation through G6PD and PFKFB3 induced glycolysis, the downstream core molecules are still unclear. It is necessary to explore the integrated and distinct mechanism. Therefore, the limitations aforementioned opens new routes for future studies to further explore therapeutics of GC.

## Conclusion

In summary, our study demonstrates that RORα inhibits gastric cancer proliferation by attenuating the glycolytic ability induced by G6PD and PFKFB3 in GC. Moreover, high proliferation and high glucose inhibited RORα expression which indicated there exist a modulation of negative feedback in GC. In addition, Deletion of RORα improved fluorouracil chemoresistance through the inhibition of glucose uptake in GC. In general, RORα might be a novel biomarker and therapeutic target for GC.

### Supplementary Information

**Additional file 1: Figure S1.** Patients profile of clinicopathological data. 162 GC patients received with CTC examination. 57 patients received chemotherapy or abandoned due to advanced disease and poor physical condition, 105 patients received with radical surgery (total or subtotal gastrectomy with D_2_ lymph node dissection) and were classified as pT_1_Nx, pT_2_Nx, pT_3_Nx and pT_4_Nx stages according to NCCN guideline. The maximum DFS time was 18 month and the follow up of patients excluded with pT_1_Nx stage. The corresponding paraffined sections of patients were collected to perform immunohistochemical staining to detect RORα, G6PD and PFKFB3 expression levels. GC, gastric cancer; CTC, circulating tumor cells, pTNM, pathology Tumor-Node-Metastasis. NCCN, national comprehensive cancer network. DFS, disease free survival.

**Additional file 2: Figure S2.** RORα deletion promotes GC proliferation induced by 3PO and DHEA in vivo The expression levels of Ki-67 and PCNA were detected through immunohistochemistry stain and MOD method in subcutaneous tumor of mice. Original 200 and 400 magnification. Scale bar = 100 μm. N = 3. The RORα-KO-MFC cells treated with 3PO (25 µM) or DHEA (250 µM) were injected into subcutaneous flank of mice until the tumor volume reached 100 to 300 mm^3^. N=3. All data are represented as the mean ± standard deviation. Vehicle was DMSO. *P < 0.05, **P < 0.01, and ***P < 0.001. Vs. RORα-KO-MFC+Vehicle group. ^+^P < 0.05, ^++^P < 0.01, and ^+++^P < 0.001. Vs. CON-MFC+Vehicle group.

**Additional file 3: Figure S3.** High proliferation and high glucose inhibit RORα expression in model of mice bearing tumor. **A** The expression levels of Ki-67 and PCNA were detected through immunohistochemistry stain and MOD method in subcutaneous tumor. Original 200 and 400 magnification. Scale bar = 100 μm. N = 3. The MFC cells treated with TGF-β1 (10 ng/ml) were injected into subcutaneous flank of mice until the tumor volume reached 100 to 300 mm^3^. N = 3. **B** RORα protein expression levels were detected by western blot in subcutaneous tumor. **C** The expression levels of RORα were detected through immunohistochemistry stain and MOD method in subcutaneous tumor. Original 200 and 400 magnification. Scale bar = 100 μm. N = 3. The MFC cells treated with glucose (5mM and 50mM) were injected into subcutaneous flank of mice until the tumor volume reached 100 to 300 mm^3^. N = 3. All data are represented as the mean ± standard deviation. Vehicle was DMSO. β-actin as a loading control. *P < 0.05, **P < 0.01, and ***P < 0.001.

**Additional file 4: Figure S4.** Inhibition of glucose uptake improved fluorouracil chemoresistance in model of mice bearing tumor. **A** Tumors were photographed and exhibited from the sacrificed mice. The MFC cells were injected into subcutaneous flank of mice until the tumor volume reached 100 to 300 mm^3^ and then fluorouracil (100 mg/kg/w) was injected into subcutaneous tissues around the tumor for 4 weeks. **B** The volume of tumors was analyzed from the sacrificed mice according to time gradient. N = 3. **C** The wight of tumors was analyzed from the sacrificed mice. N = 3. All data are represented as the mean ± standard deviation. Vehicle was DMSO. *P < 0.05, **P < 0.01, and ***P < 0.001.

## Data Availability

The data that support the findings of this study are available on request from the corresponding author, [Zhengguang Wang], upon reasonable request.
