# Supplementary figures and images for: RORα inhibits gastric cancer proliferation through attenuating G6PD and PFKFB3 induced glycolytic activity
Source: Cancer Cell Int. 2024 Jan 6;24:12. doi: 10.1186/s12935-023-03201-4 (PMC10770990; doi:10.1186/s12935-023-03201-4)

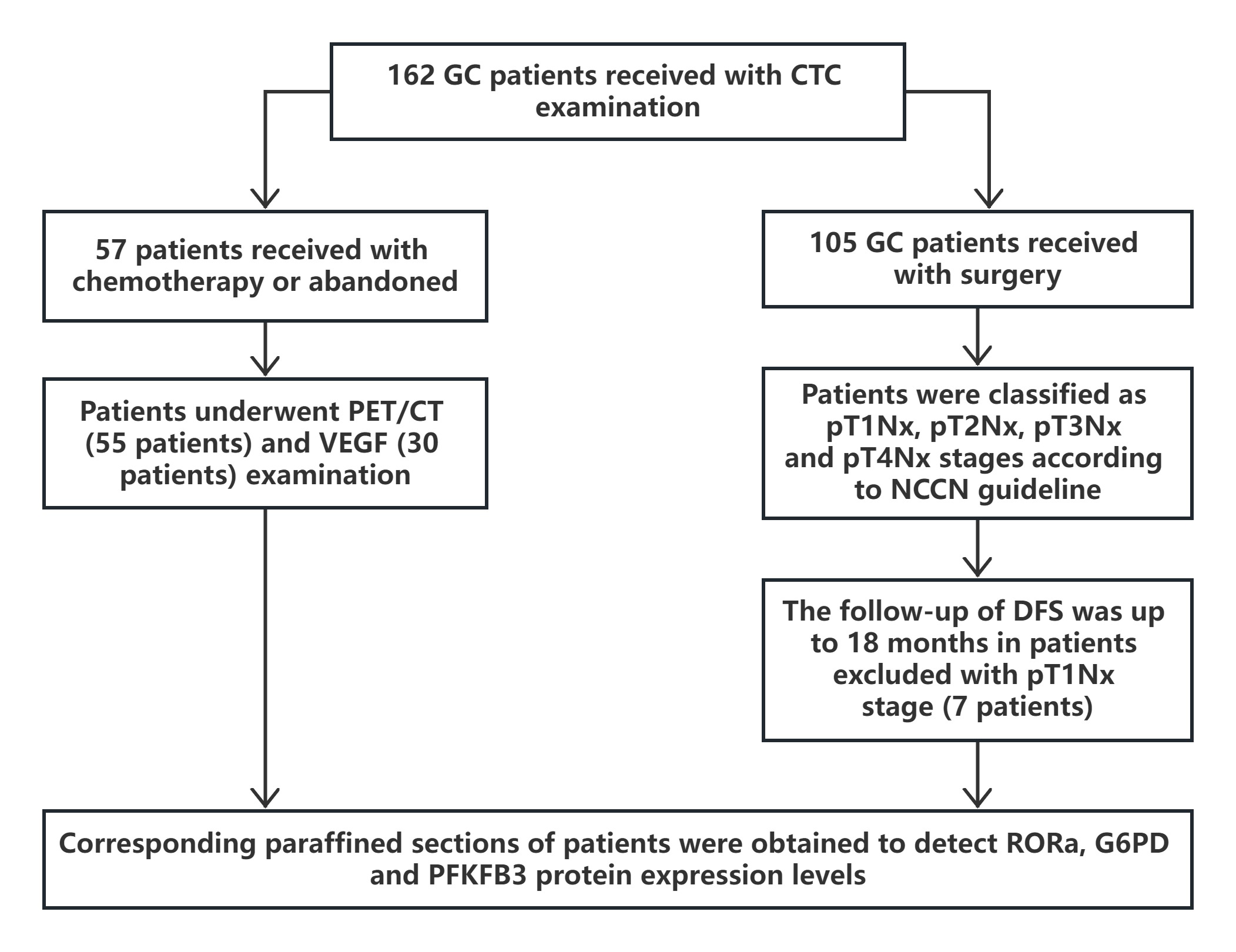

Supplement: Supplementary file 1 — Additional file 1: Figure S1. Patients profile of clinicopathological data. 162 GC patients received with CTC examination. 57 patients received chemotherapy or abandoned due to advanced disease and poor physical condition, 105 patients received with radical surgery (total or subtotal gastrectomy with D2 lymph node dissection) and were classified as pT1Nx, pT2Nx, pT3Nx and pT4Nx stages according to NCCN guideline. The maximum DFS time was 18 month and the follow up of patients excluded with pT1Nx stage. The corresponding paraffined sections of patients were collected to perform immunohistochemical staining to detect RORα, G6PD and PFKFB3 expression levels. GC, gastric cancer; CTC, circulating tumor cells, pTNM, pathology Tumor-Node-Metastasis. NCCN, national comprehensive cancer network. DFS, disease free survival. [file 12935_2023_3201_MOESM1_ESM.jpg]

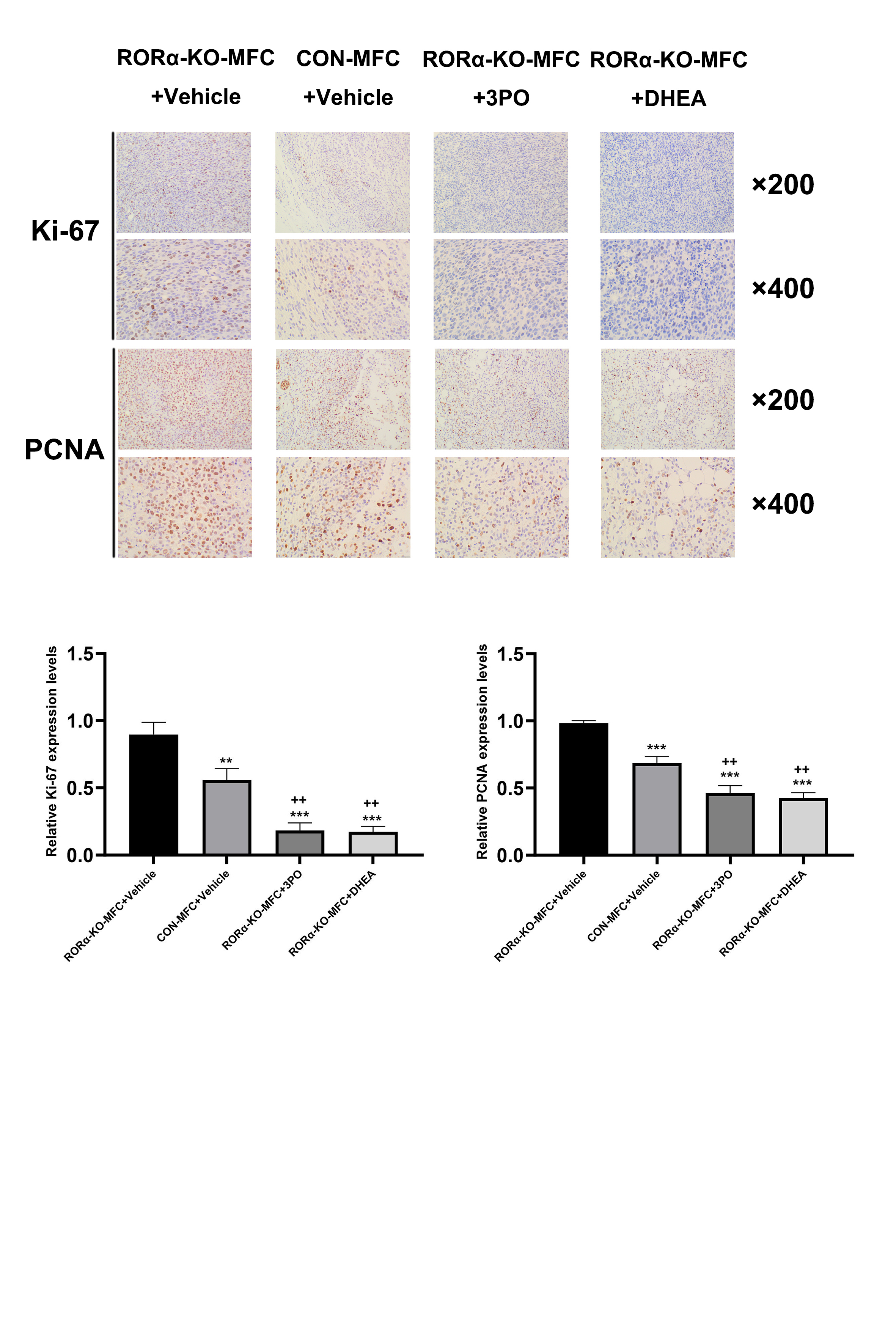

Supplement: Supplementary file 2 — Additional file 2: Figure S2. RORα deletion promotes GC proliferation induced by 3PO and DHEA in vivo The expression levels of Ki-67 and PCNA were detected through immunohistochemistry stain and MOD method in subcutaneous tumor of mice. Original 200 and 400 magnification. Scale bar = 100 μm. N = 3. The RORα-KO-MFC cells treated with 3PO (25 µM) or DHEA (250 µM) were injected into subcutaneous flank of mice until the tumor volume reached 100 to 300 mm3. N=3. All data are represented as the mean ± standard deviation. Vehicle was DMSO. *P < 0.05, **P < 0.01, and ***P < 0.001. Vs. RORα-KO-MFC+Vehicle group. +P < 0.05, ++P < 0.01, and +++P < 0.001. Vs. CON-MFC+Vehicle group. [file 12935_2023_3201_MOESM2_ESM.jpg]

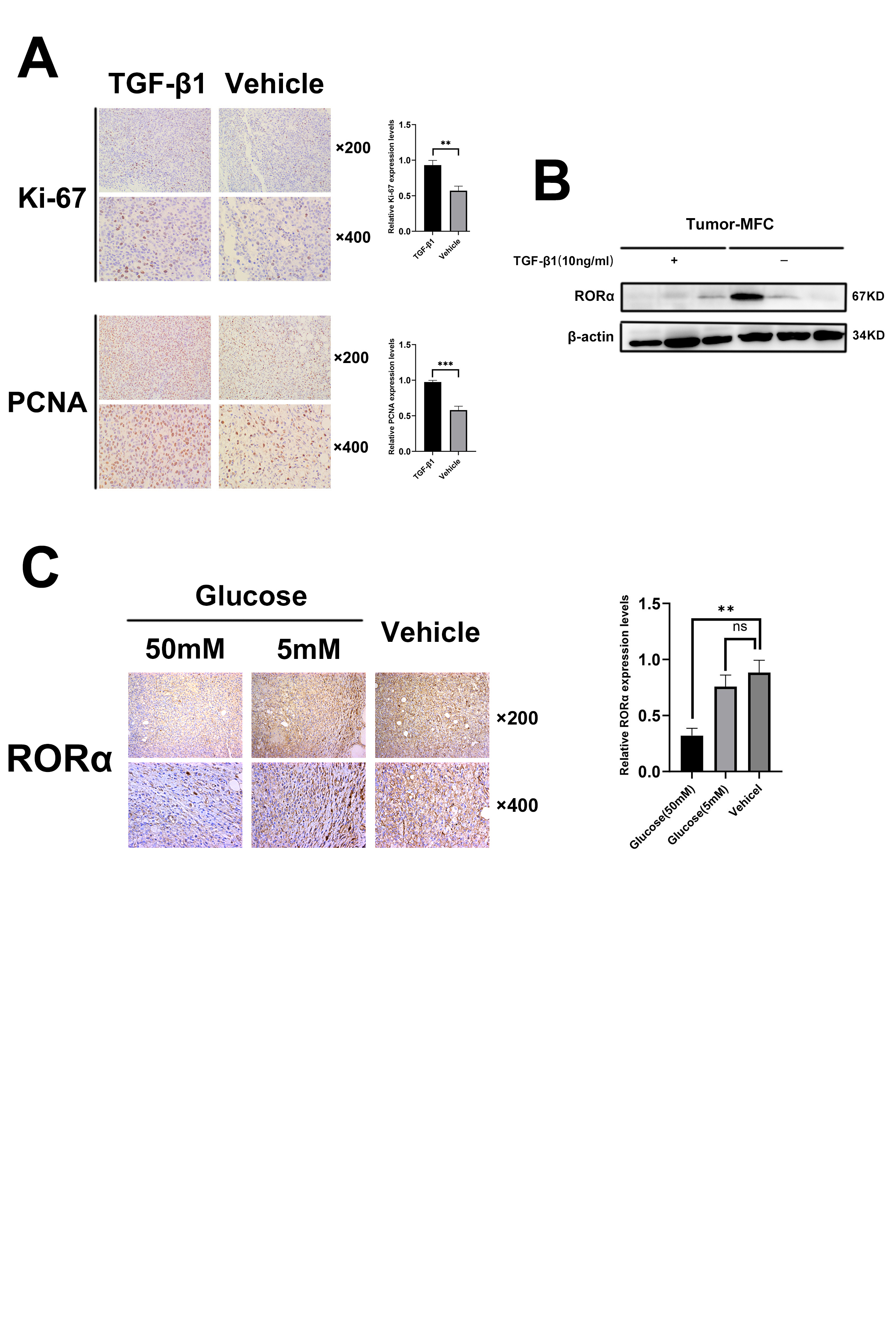

Supplement: Supplementary file 3 — Additional file 3: Figure S3. High proliferation and high glucose inhibit RORα expression in model of mice bearing tumor. A The expression levels of Ki-67 and PCNA were detected through immunohistochemistry stain and MOD method in subcutaneous tumor. Original 200 and 400 magnification. Scale bar = 100 μm. N = 3. The MFC cells treated with TGF-β1 (10 ng/ml) were injected into subcutaneous flank of mice until the tumor volume reached 100 to 300 mm3. N = 3. B RORα protein expression levels were detected by western blot in subcutaneous tumor. C The expression levels of RORα were detected through immunohistochemistry stain and MOD method in subcutaneous tumor. Original 200 and 400 magnification. Scale bar = 100 μm. N = 3. The MFC cells treated with glucose (5mM and 50mM) were injected into subcutaneous flank of mice until the tumor volume reached 100 to 300 mm3. N = 3. All data are represented as the mean ± standard deviation. Vehicle was DMSO. β-actin as a loading control. *P < 0.05, **P < 0.01, and ***P < 0.001. [file 12935_2023_3201_MOESM3_ESM.jpg]

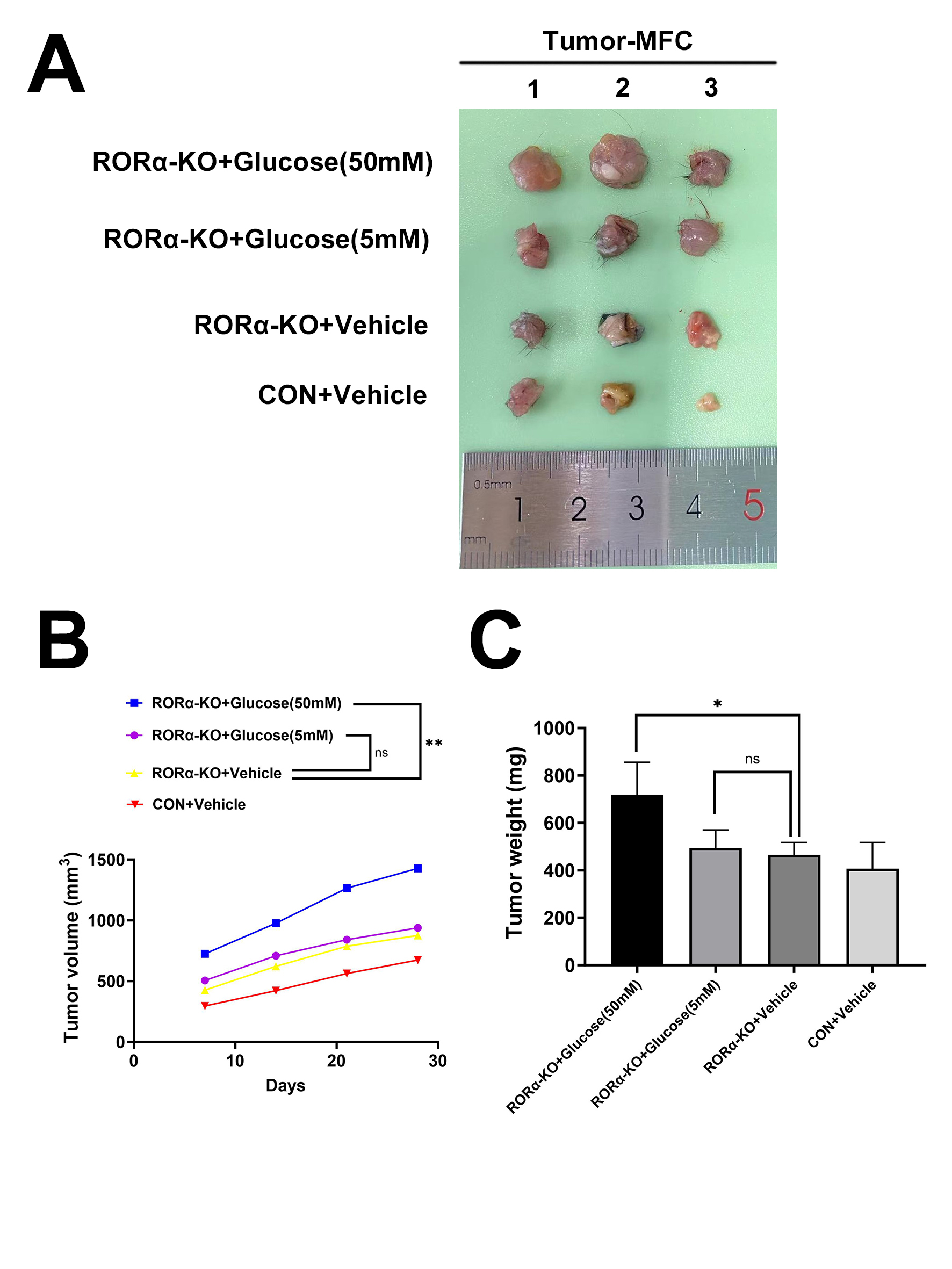

Supplement: Supplementary file 4 — Additional file 4: Figure S4. Inhibition of glucose uptake improved fluorouracil chemoresistance in model of mice bearing tumor. A Tumors were photographed and exhibited from the sacrificed mice. The MFC cells were injected into subcutaneous flank of mice until the tumor volume reached 100 to 300 mm3 and then fluorouracil (100 mg/kg/w) was injected into subcutaneous tissues around the tumor for 4 weeks. B The volume of tumors was analyzed from the sacrificed mice according to time gradient. N = 3. C The wight of tumors was analyzed from the sacrificed mice. N = 3. All data are represented as the mean ± standard deviation. Vehicle was DMSO. *P < 0.05, **P < 0.01, and ***P < 0.001. [file 12935_2023_3201_MOESM4_ESM.jpg]
